# The Level of Serum Pepsinogen in Diagnosing and Evaluating the Severity of Subacute Combined Degeneration Due to Vitamin B12 Deficiency

**DOI:** 10.3389/fneur.2021.604523

**Published:** 2021-03-19

**Authors:** Xiaoyan Chen, Rong Wang, Xusheng Huang, Fei Yang, Shengyuan Yu

**Affiliations:** ^1^Department of Neurology, First Medical Center of Chinese PLA General Hospital, Beijing, China; ^2^General Hospital of Taiyuan Iron and Steel (Group, Co., Ltd.) Shanxi, China

**Keywords:** subacute combined degeneration, vitamin B12 deficiency, pepsinogen, PG1/2 ratio, chronic autoimmnue atrophic gastritis

## Abstract

Subacute combined degeneration (SCD) is a neurological complication of cobalamin deficiency, which is usually caused by chronic autoimmune atrophic gastritis. Serum pepsinogen 1 and the ratio of pepsinogen 1/pepsinogen 2 (PG1/2) can reflect the severity of gastric atrophy.

**Objective:** This work aims to investigate whether decreased serum PG1 and PG1/2 ratio are helpful in diagnosing SCD and reflecting the severity of SCD.

**Methods:** We retrospectively analyzed the clinical and laboratory tests of 65 cases of SCD due to vitamin B12 deficiency and compared the laboratory parameters of SCD with 65 age- and sex-matched amyotrophic lateral sclerosis (ALS) patients.

**Results:** PG1 and PG1/2 ratio were decreased in 80 and 52.3% of SCD patients, respectively. Compared to patients with PG1/2 ratio ≥3.0, patients with PG1/2 ratio <3.0 had more severe anemia, larger mean corpuscular volume (MCV), lower level of vitamin B12, higher folate and homocysteine (Hcy), more severe changes in somatosensory evoked potential (SEP), and higher rate of lesions in spinal MRI (*P* < 0.05). PG1 and PG1/2 ratio had inverse correlation with MCV and N20 latency in SEP examination (*P* < 0.05). PG1/2 ratio, RBC count, and Hcy were independent risk factors for SCD in logistic regression analyses. The ROC curve analysis revealed that the diagnostic accuracy of PG1 and PG1/2 ratio was 72.2 and 73.0%, respectively, while the cutoff values were 22.4 ng/ml and 2.43 for SCD, respectively.

**Conclusions:** Decreased PG1 and PG1/2 ratio are helpful for the diagnosis and evaluation of the severity of SCD due to vitamin B12 deficiency.

## Introduction

Subacute combined degeneration is characterized by myelopathy of the posterior and lateral columns and peripheral neuropathy due to cobalamin (vitamin B12) deficiency and occasionally would be accompanied by optic neuropathy and encephalopathy (1, 2). Neurological deficit is reversible with cobalamin treatment at the early stage of disease, but residual neurological abnormalities would persist with delayed treatment. Therefore, early diagnosis and standardized treatment are essential for the prognosis.

However, patients were not always diagnosed correctly without a thorough workup by experienced doctors. The symptoms would be atypical. The laboratory findings of decreased plasma cobalamin level and megaloblastic anemia are helpful in diagnosing SCD, but the serum cobalamin level would be normal or even elevated (3, 4), perhaps due to interference of anti-intrinsic factor antibodies in measuring cobalamin (5), overgrowth of intestinal bacteria which produced cobalamin analogs (6), or cobalamin administration before diagnosis (7). Elevated homocysteine (Hcy) or methylmalonic acid level can be used as indirect evidence of cobalamin deficiency, but the level of Hcy or methylmalonic acid would also be influenced by many other factors apart from cobalamin deficiency, and they would be normal in some cobalamin-deficient patients (2). Besides that, the laboratory test of methylmalonic acid is not available in many hospitals in China. Megaloblastic anemia was also absent in some patients with cobalamin deficiency, and neurological manifestations would precede hematological abnormalities (4, 8, 9). Furthermore, the spinal MRI with lesions typically in the dorsal and lateral columns only had a sensitivity of diagnosing SCD that ranged from 14.8 to 52.8% (4, 10, 11).

Cobalamin in food is bound to animal protein. Cobalamin deficiency can be caused by an inadequate intake of animal foods, gastrointestinal malabsorption, transporting disorders caused by gene mutations, inactivation of methylcobalamin by inhaling laughing gas, etc. (2). Cobalamin malabsorption by the gastrointestinal tract is the most common cause of non-dietary-induced cobalamin deficiency disease. In the stomach, cobalamin is separated from its binding protein by peptic digestion and bound to R-proteins. Then, the cobalamin–R-protein complex is split by pancreatic enzymes in the duodenum where cobalamin is bound to intrinsic factor (IF). The cobalamin–IF complex binds to the ileal receptors in the terminal ileum (12). The most frequent cause of clinically presented cobalamin deficiency is loss of IF due to chronic autoimmune atrophic gastritis (CAAG) (13), which is an autoimmune disease affecting the corpus–fundus mucosa of the stomach (14). Positive parietal cell antibodies (PCA) and antibodies to the IF (anti-IF) are markers of CAAG (14). In patients with CAAG, hydrochloric acid, pepsin, and IF are all impaired, resulting in cobalamin malabsorption. The proenzyme of pepsin is pepsinogen (PG), which contains two main groups: PG1 and PG2. Both PG1 and PG2 are produced by the chief cells of the gastric body and fundus, while PG2 is also produced by other cells such as chief cells of the oxyntic gland areas, the mucosa neck cells in the gastric fundus, the cardiac gland, the pyloric gland cells in the gastric antrum, Brunner's glands in the proximal duodenum, and the prostate gland (15). The combination of serum pepsinogen1 (PG1) below 70 ng/ml and PG1/2 ratio below three has been considered to be a serum biomarker to diagnose gastric atrophy (16–18).

Whether decreased serum pepsinogen is helpful in diagnosing SCD and whether the level of pepsinogen in the serum would be associated with SCD severity have not been investigated yet. Therefore, this study was performed to try to answer the questions.

## Materials and Methods

### Participants

A total of 65 in-patients diagnosed with SCD between January 2014 and June 2019 in our department were retrospectively analyzed. The inclusion criteria were as follows: 1) diagnostic criteria (4) of SCD fulfilling (a) clinical symptoms and signs of posterior and lateral columns or peripheral nerve impairment, (b) evidence of vitamin B12 deficiency [low serum vitamin B12 level and/or elevated Hcy, increased mean corpuscular volume (MCV) or clinical improvement after regular vitamin B12 treatment] and 2) all the patients were tested with serum anti-IF, PG1, PG2, and PG1/2 ratio after hospital admission. Patients with SCD and mimics associated with decreased serum copper (19), inhaling laughing gas (20) and other non-B12 deficiency myelopathy (e.g., syphilitic myelitis, hepatic myelopathy, spinal inflammatory demyelination) were excluded from this group of patients.

In order to evaluate the diagnostic value of pepsinogen for SCD, we included 65 cases of amyotrophic lateral sclerosis (ALS) patients, with comparable age and sex and who were admitted to our department during the same period, as controls. Health controls were not included in this study because pepsinogen and PG1/2 ratio were not routinely measured in the physical examination center. The diagnostic criteria of ALS were referred to the revised El Escorial criteria (21), and all the 65 patients underwent serum pepsinogen test. None of the SCD or ALS patients should have had stroke, diabetes mellitus, severe heart, liver, or kidney diseases, untreated cancer, or other neurological diseases which would influence the limb or brain function. This study was approved by the Ethics Committee of Chinese PLA General Hospital.

### Clinical Evaluation and Auxiliary Examination

For SCD patients, detailed information of history and physical examination were collected. The functional disability scale of SCD (22) and the electrophysiological tests for each patient were recorded. For both SCD and ALS patients, demographic data and laboratory tests of red blood cell (RBC) counts, hemoglobin (42), MCV, vitamin B12, folate, Hcy, anti-IF, PG1, PG2, and PG1/2 ratio were recorded.

### Statistical Analysis

The statistical analyses were performed using SPSS 20.0 (Chicago, IL, USA) and MedCalc Statistical Software version 15.8 (MedCalc Software bvba, Ostend, Belgium; https://www.medcalc.org; 2015). Numerical data were presented as mean ± SD if normally distributed or presented as median value (interquartile range, IQR) if not normally distributed. Categorical data were presented as frequencies with absolute numbers and percentages. Independent-samples *t*-tests were used to compare continuous data if normally distributed, while Kruskall–Wallis tests were applied when the continuous data were not normally distributed. Chi-square tests were used to compare categorical data. Pearson correlation tests were performed if the data were continuous and normally distributed; otherwise, Spearman correlation tests were used. Binary logistic regression analysis was used to examine the relationship between laboratory parameters and the diagnosis of SCD. Receiver operating characteristic (ROC) curves using MedCalc Statistical Software were constructed to extract the diagnostic accuracy and corresponding cutoff values for the respective groups. The cutoff values from each evaluation were used to determine sensitivity and specificity. The ROCs for each laboratory parameter were compared between SCD and ALS patients. The variable was considered as significant if the two-tailed *P*-value was <0.05.

## Results

### Demographic and Clinical Characteristics of SCD Patients

A total of 65 (47 males and 18 females) SCD patients, aged 58.54 ± 14.18 years, fulfilled the inclusion criteria in this study. Disease duration ranged from 20 days to 240 months, with the median (IQR) value as 12 (3.5–24) months. The probable causes according to past history were vegetarian—six (9.2%), inadequate food intake—five (7.7%), partial gastrectomy—one (1.5%), gastritis and/or gastric ulcer-−17 (26.2%), and unknown-−39 (60%). Other past history included anemia—seven (10.8%), thyroid dysfunction—six (9.2%), recurrent oral ulcer—two (3.1%), connective tissue diseases—two (3.1%), bronchitis—two (3.1%), eczema—one (1.5%), hypertension −15 (23.1%), and tumor surgery—two (3.1%). Only seven (10.8%) patients had ever been diagnosed as SCD. After a confirmative diagnosis of SCD in our department, all the patients received regular intramuscular methylcobalamin treatment and reported improvement during follow-up at 3 or 6 months.

**Figure 1:**
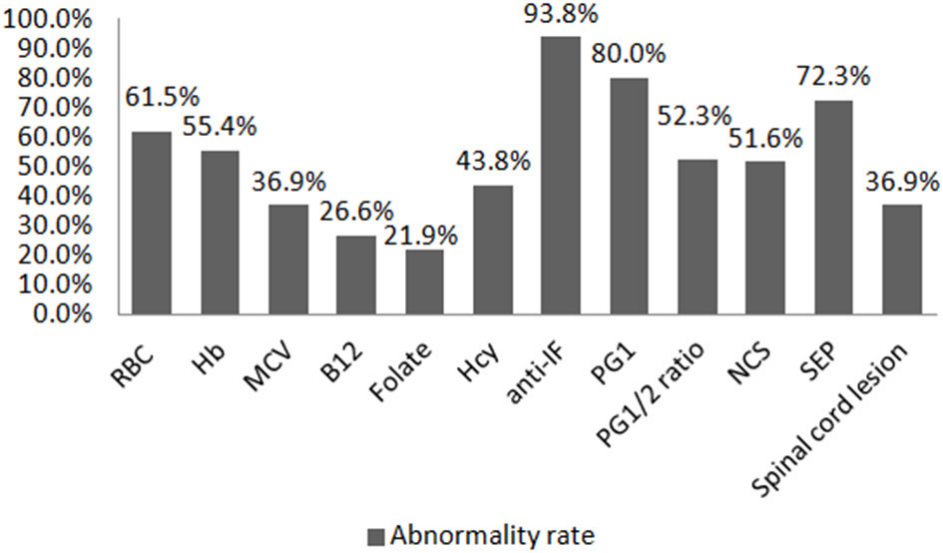
Abnormality rate of different auxiliary examinations. RBC, red blood cell; Hb, hemoglobin; MCV, mean corpuscular volume; B12, vitamin B12; Hcy, homocysteine; anti-IF, antibody to the intrinsic factor; PG1, pepsinogen 1; PG1/2, pepsinogen 1/pepsinogen 2; NCS, nerve conduction study; SEP, somatosensory evoked potential. The abnormality rates of B12 and folate only refer to the rates of decreased value in this figure.

Thirty-two (49.2%) SCD patients had taken cobalamin treatment before hospitalization (either oral or intramuscular methylcobalamin or adenosylcobalamin regularly or irregularly for 3 days to several months), and 21 (32.3%) had taken some medication, but it was unclear whether such contained cobalamin, while only 12 (18.5%) were untreated. The B12 level after admission was found to be elevated in 19/64 (29.7%) patients (16 had been treated with cobalamin: seven orally and nine intramuscularly; three were unclear of what drugs were taken). The B12 value was decreased in 17/64 (26.6%) patients, of whom seven had been treated with cobalamin (four by oral and three by intramuscular administration). The serum folic acid value was elevated in 22/64 (34.4%) patients and decreased in 14/64 (21.9%) patients. None of the patients reported whether they had taken folic acid before hospitalization. Hcy was elevated in 28/64 (43.8%) patients. The red blood cell count and Hb were decreased in 40/65 (61.5%) and 36/65 (55.4%) patients, respectively. MCV was increased in 24/65 (36.6%) patients. The anti-IF was positive in 61/65 (93.8%) patients. The PG1 and PG1/2 ratio were decreased in 52/65 (80.0%) and 34/65 (52.3%) patients, respectively. Both PG1 and PG1/2 ratio were decreased in 52.3% patients. The abnormality rate of nerve conduction study (NCS) and somatosensory evoked potential (SEP) was 32/62 (51.6%) and 47/59 (72.3%), respectively. Only 24/58 (36.9%) patients had lesions in the spine as confirmed by MRI (Figure 1). Only 29 (44.6%) patients underwent gastroscopic examination, and all were reported as chronic gastritis, of which eight had biopsy suggesting chronic inflammation and two were found to have *Helicobacter pylori* infection.

### Relationship of Serum PG1 Level, PG1/2 Ratio With the Clinical Data of SCD Patients

Compared with patients of PG1/2 ratio ≥3.0, patients of PG1/2 ratio <3.0 were relatively younger (*P* = 0.007), demonstrating a significantly lower RBC count (*P* < 0.001), Hb (*P* = 0.005), B12 level (*P* = 0.024), serum PG1 value (*P* < 0.001), and PG2 value (*P* < 0.001), relatively larger MCV (*P* < 0.001), and higher value of serum folic acid (*P* < 0.001) and Hcy (*P* = 0.012). The proportion of patients with absent P1 SEP was much higher in patients of PG1/2 ratio <3.0 compared with those of PG1/2 ratio≥3.0 (69.0 vs. 20%, *P* < 0.001). Bilateral N20 latency was much longer in patients of PG1/2 ratio <3.0 than ≥3.0 (left N20 latency, ms: 22.56 ± 2.09 vs. 20.62 ± 1.48*, P* = 0.002; right N20 latency, ms: 22.53±2.07 vs. 20.59±1.54; *P* = 0.002). Significantly more patients had lesions in the spinal cord in patients of PG1/2 ratio <3.0 than ≥3.0 (59.4 vs. 19.2%, *P* = 0.002). There were no differences in disease duration, functional disability scale of SCD (either total score or sub-item scores), serum anti-IF value or positive rate of anti-IF, abnormality rate of NCS or SEP, rate of absent wave in motor or sensory nerve conduction, mean value of motor nerve conduction velocity and the amplitude of compound muscle action potential, mean value of sensory nerve conduction velocity, and sensory nerve action potential between patients of PG1/2 ratio <3.0 and ≥3.0 (Table 1).

**Table 1:** Clinical variables of SCD subgroups with normal or abnormal PG1/2 ratio.

**Items**		**PG1/2 ratio <3.0 (*N* = 34)**	**PG1/2 ratio 3.0 (*N* = 31)**	***P***
Age		54.12 ± 14.07	63.39 ± 16.83	0.007
Gender (M/F)		22/12	25/6	0.176
Duration (month)		6.0 (2.75–21.75)	13.0 (8.0–32.0)	0.116
RBC (10^12^/L)		3.36 ± 0.86	4.30 ± 0.68	<0.001
Hb (g/L)		118.12 ± 24.83	134.03 ± 18.89	0.005
MCV (fl)		103.66 ± 9.07	92.65 ± 6.45	<0.001
Vit B12 (pg/mL)		235.2 (50.0–1397.0)	625.05 (414.28–1263.75)	0.024
Folate (ng/ml)		16.26 (11.09–20.0)	5.57 (3.64–9.52)	<0.001
Hcy (μmol/L)		27.80 (11.8–62.85)	12.9 (10.3–21.0)	0.012
Anti-IF (AU/ml)		14.86 (4.43–57.24)	12.6 (10.53–14.06)	0.400
PG1 (ng/ml)		4.1 (2.7–5.53)	66.0 (44.7–88.1)	<0.001
PG2 (ng/ml)		5.5 (3.8–6.88)	9.2 (6.8–14.2)	<0.001
PG1/2 ratio		0.75 (0.51–1.06)	6.73 (4.75–8.07)	<0.001
NCS (*N* = 62)	Abnormal (N)	16	16	0.805
	Absent wave (N)	6	4	0.728
	Normal (N)	16	14	
SEP (*N* = 59)	Abnormal (N)	26	21	0.061
	Absent wave (N)	20	6	<0.001
	Normal (N)	3	9	
MRI lesion (*N* = 58)	Visible lesion (N)	19	5	0.002
	No visible lesion (N)	13	21	

*SCD, subacute combined degeneration; M/F, male/female; RBC, red blood cell; Hb, hemoglobin; MCV, mean corpuscular volume; Hcy, homocysteine; Anti-IF, antibody to the intrinsic factor; PG1/2, pepsinogen 1/pepsinogen 2; NCS, nerve conduction study; SEP, somatosensory evoked potential; N, number*.

**Figure 2:**
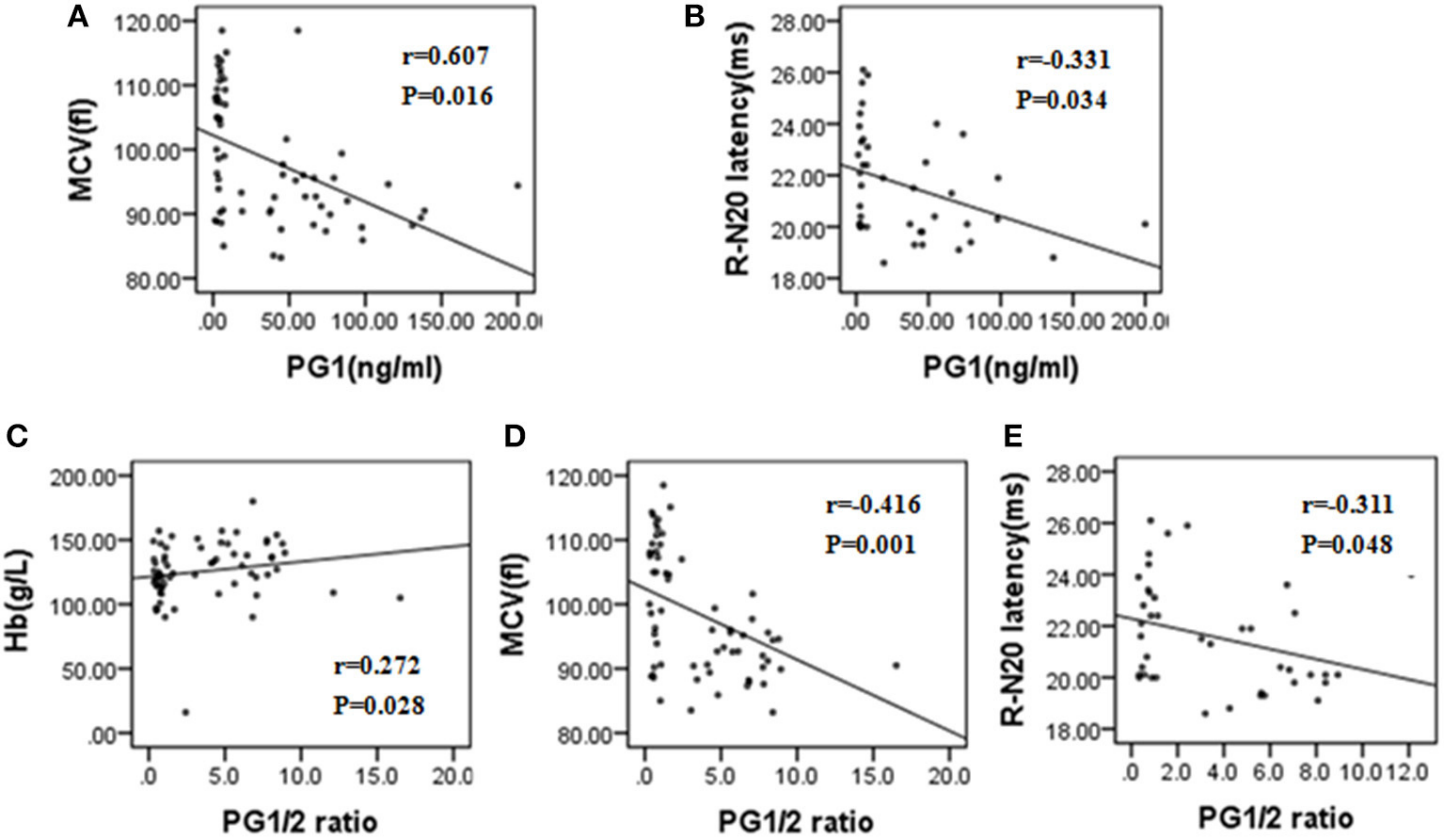
**(A–E)** Correlation analysis of PG1, PG1/2 ratio with other laboratory tests and electrophysiological parameters in patients with subacute combined degeneration. MCV, mean corpuscular volume; Hb, hemoglobin; R-N20, right N20 in somatosensory evoked potential test; PG1, pepsinogen 1; PG1/2, pepsinogen 1/pepsinogen.

In the Spearman correlation analysis (two-tailed), the PG1/2 ratio showed a positive correlation with Hb (*r* = 0.272, *P* = 0.028) but a negative correlation with MCV (*r* = −0.416, *P* = 0.001) and right N20 latency of SEP test (*r* = −0.311, *P* = 0.048). PG1 also had a positive correlation with RBC count (*r* = 0.607, *P* = 0.016) and a negative correlation with right N20 latency of SEP test (*r* = −0.331, *P* = 0.034) (Figure 2).

### Diagnostic Value of PG1 and PG1/2 Ratio in Diagnosing SCD

In comparison with the age- and sex-matched ALS patients, the SCD patients had a relatively lower RBC count (*P* <0.001), Hb (*P* = 0.001) but larger MCV (*P* < 0.001). Serum folic acid (*P* = 0.009) and Hcy (*P* = 0.004) were relatively higher, and B12 tended to be lower without reaching statistical significance (*P* = 0.051) in SCD patients. The values of PG1, PG2, and PG1/2 ratio were all relatively lower in SCD patients than in ALS patients (*P* < 0.001) (Table 2). The number of patients was adequate to perform the logistic regression analysis, in which only PG1/2 ratio (*b* = −0.212, *P* = 0.005), RBC count (*b* = −1.069, *P* = 0.006), and Hcy (*b* = 0.059, *P* = 0.009) were independent risk factors for SCD, and we got an equation as log (P) = 4.735–1.069 × RBC - 0.212 × PG1/2 ratio + 0.059 × Hcy.

**Table 2:** Demographics and Laboratory tests of SCD and ALS patients.

	**SCD(*N*=65)**	**ALS(*N*=65)**	***P***
Age	58.54 ± 14.18	56.55 ± 8.87	0.341
Gender(M/F)	47/18	47/18	1.000
RBC counting (10^12^/L)	3.81 ± 0.91	4.50 ± 0.48	<0.001
Hb(g/L)	125.71 ± 23.44	137.85 ± 14.50	0.001
MCV(fl)	98.41 ± 9.63	90.61 ± 5.13	<0.001
Vit B12(pg/mL)	507.55(86.13–1235.75)	890.45(562.65–1379.5)	0.051
Folic acid(ng/ml)	11.30 ± 6.27	8.07 ± 4.03	0.009
Hcy(umol/L)	17.20(10.43–50.38)	11.00(7.88–14.85)	0.004
Pepsinogen 1(ng/ml)	8.00(3.85–63.30)	54.80(43.55–75.80)	0.005
Pepsinogen 2(ng/ml)	6.80(5.20–9.50)	10.40(6.40–14.30)	<0.001
PG1/2 ratio	1.67(0.74–6.59)	5.56(4.34–7.98)	<0.001

*SCD, subacute combined degeneration; ALS, amyotrophic lateral sclerosis; M/F, male/female; RBC, red blood cell; Hb, hemoglobin; MCV, mean corpuscular volume; Hcy, homocysteine; PG1/2, pepsinogen 1/pepsinogen 2*.

**Figure 3:**
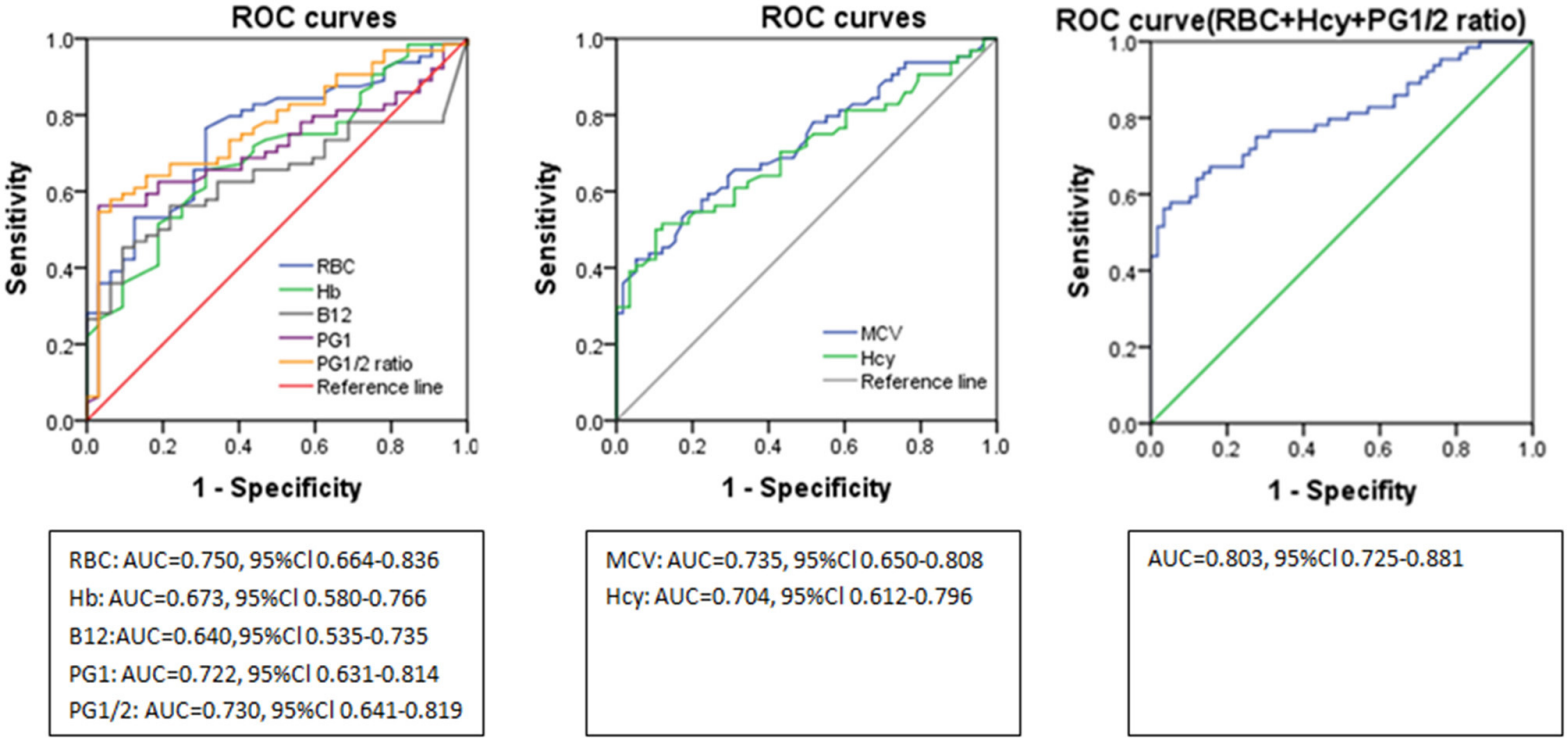
ROC curves of the laboratory parameter in distinguishing subacute combined disorder from amyotrophic lateral sclerosis. ROC, receiver operating characteristic; RBC, red blood cell; Hb, hemoglobin; PG1/2, pepsinogen 1/pepsinogen 2; MCV, mean corpuscular volume; Hcy, homocysteine; AUC, area under the curve.

ROC curve analyses indicated the diagnostic accuracy as follows: RBC count (AUC = 0.750), Hb (AUC = 0.673), MCV (AUC = 0.735), B12 (AUC = 0.640), Hcy (AUC = 0.704), PG1 (AUC = 0.722), and PG1/2 ratio (AUC = 0.730). The diagnostic accuracy of PG1 and PG1/2 ratio was equal to those of RBC count, MCV, and Hcy, which was between 0.7 and 0.8. The combined diagnostic accuracy of RBC + Hcy + PG1/2 ratio was 0.803, which was much higher than those of Hb (*P* = 0.004), Hcy (*P* = 0.03), and vitamin B12 (*P* < 0.001) (Figure 3 and Table 3). In the ROC curve analyses, MCV, Hcy, PG1, and PG1/2 all had good specificity as high as over 89% but lower sensitivity as 43.1–55.4%. The value of MCV >98.3 fl and Hcy >17.8 umol/L had very good specificity for diagnosing SCD. The cutoff values of PG1 and PG1/2 ratio were 22.4 ng/ml and 2.43. When the PG1 value was 70 ng/ml, the sensitivity reached to 80%, but the specificity decreased to 33.8%. When PG1/2 was 3.0, the sensitivity was 53.8%, and the specificity was 95.4%. Actually, all the patients with PG1/2 <3.0 had PG1 <70 ng/ml in our study.

**Table 3:** Parameters of ROC curves in distinguishing subacute combined disorder from amyotrophic lateral sclerosis.

	**AUC**	**95%Cl**	**P**	**Cut-off value**	**Sensitivity**	**Specificity**
RBC(× 10^12^/L)	0.750^a^	0.664–0.836	<0.001	4.44	76.9%	67.7%
Hb(g/L)	0.673	0.580–0.766	0.001	133.5	61.5%	67.7%
MCV(fl)	0.735^b^	0.650–0.808	<0.001	98.3	43.1%	95.4%
B12(pg/mL)	0.640	0.535–0.735	0.01	423	45.3%	90.62%
Hcy(umol/L)	0.704	0.612–0.796	<0.001	17.80	50%	89.7%
PG1(ng/ml)	0.722	0.631–0.814	<0.001	22.4	55.4%	95.4%
PG1/2 ratio	0.730^c^	0.641–0.819	<0.001	2.43	52.3%	98.5%
RBC+Hcy+PG1/2	0.803^d^	0.725–0.881	<0.001	0.68	56.3%	96.6%

a *The AUC of RBC was significantly bigger than that of Hb(P=0.003)*;

b *The AUC of PG1/2 ratio was bigger than that of B12(P=0.01)*;

c *The AUC of PG1/2 ratio was bigger than that of B12(P = 0.03)*;

d *The AUC of RBC+Hys+PG1/2 was significantly bigger than that of Hb(P = 0.004), B12(P <0.001), Hyc(P=0.03). ROC, receiver operating characteristic; AUC, area under the curve; RBC, red blood cell; Hb, hemoglobin; MCV, mean corpuscular volume; Hcy, homocysteine; PG1/2, pepsinogen 1/pepsinogen 2*.

## Discussion

To our knowledge, this is the first study to investigate the role of pepsinogen in SCD. We found that a low level of PG1/2 ratio would be helpful in diagnosing SCD due to vitamin B12 deficiency and could reflect the severity of hematological and neurological involvement, whereas the commonly used laboratory evidence of vitamin B12 deficiency, such as low level of serum vitamin B12, macrocytosis, and hyperhomocysteinemia, would usually be lacking as no more than half of our patients demonstrated positive findings in this evidence.

SCD is caused by B12 deficiency, while severe vitamin B12 deficiency is most commonly caused by CAAG (13). CAAG is a multifaceted disease which would manifest as unspecific or subtle gastrointestinal symptoms and other disorders related to B12 deficiency such as bone marrow hypofunction, hyperhomocysteinemia, spinal cord degeneration with nerve demyelination, and infertility or recurrent miscarriage that would lead the patient to consult different specialist physicians, causing a substantial diagnostic delay (23, 24). Promotion of awareness of this disease and the resulting clinical spectrum is needed by physicians in relevant fields. As auto-immune markers of CAAG, PCA was reported to be positive in 85–90% of patients with suboptimal specificity, whereas anti-IF was reported to have high specificity (98.6%) and relatively low sensitivity (60%) in CAAG patients (14). Since the measurement of PCA was unavailable in our hospital or in institutions around in previous years, very few patients in our database had a PCA test. Therefore, we did not take PCA into analysis in this study. Anti-IFs were measured in all the 65 included SCD patients. The positive rate of anti-IF in our study was as high as 93.8%, which indicated that most of the SCD patients were caused by CAAG. Although we could not exclude false positivity of anti-IF in our study and we could not compare the positive rate of anti-IF between SCD patients and ALS patients as ALS patients did not regularly have anti-IF test, the high positive rate of anti-IF did provide evidence of autoimmune gastritis as the main cause of SCD in this study since the positive rate was only 4.63% in the general Chinese population (25). Rarer causes were being vegetarian, inadequate food intake, and partial gastrectomy. Positive anti-IF was helpful in diagnosing SCD, but it could not reflect disease severity in our study.

Low serum vitamin B12 level is the direct evidence of B12 deficiency, but it has low sensitivity and specificity (26). In our study, only 26.6% of SCD patients showed decreased serum B12. B12 level was found to be decreased in 54.4–69.6% of SCD patients in another two case series (4, 27). Such a low rate of decreased B12 and a high rate of normal or elevated B12 in our study would be largely due to medication interference since at least 49.2% of SCD patients had received B12 supplementation before hospitalization. On the other hand, B12 over the lower limit of normal ranges does not exclude B12 deficiency. Higher levels (but not more than 350 pg/ml) were recorded in healthy elderly patients with increased serum methylmalonic acid, suggestive of a deficiency state (3, 28). The true lower limits of normal B12 would be further defined. In our study, B12 lower than 423 pmol/L had 90.62% specificity in distinguishing SCD from ALS patients, but this result should be cautiously elucidated as the value was interfered by B12 supplementation. Furthermore, the presence of anti-IF would interfere with the B12 assay using an intrinsic factor binding method, resulting in spuriously normal vitamin B12 levels (26). Therefore, a more accurate cutoff value of defining B12 deficiency for SCD needs more studies, in which patients should not take B12 supplementation before having serum B12 test, and the B12 test should not use the general intrinsic factor binding method.

B12 is required for folate conversion from methylfolate to tetrahydrofolate, which is transferred to Hcy to form methionine. Therefore, B12 and/or folate deficiency can result in Hcy elevation (26). In our study, 34.4% of SCD patients had elevated folate, and the mean value of folate was significantly higher than that of controlled ALS patients. This is similar to previous reports that 20–30% of patients with cobalamin deficiency had elevated serum folate, and neurologically affected patients had higher folate levels than unaffected ones (29). The elevated folate is largely trapped as methylfolate following an impairment of methionine synthase activity (29). More evidences in the literature showed that patients with low B12 and high folate levels had higher levels of Hcy, lower Hb, and more common cognitive decline (30, 31). Individuals with low serum B12 and high serum folate had lower holotranscobalamin, indicating a depletion of circulating active B12 when serum folate was high (32). In our study, SCD patients with PG1/2 ratio <3, compared to those with PG1/2 ratio ≥3.0, had significantly higher Hcy, higher folate, and lower B12 level in the serum, supporting that relatively elevated folate would be associated with even more severe B12 deficiency. There are further evidences that high folate intake would exacerbate conditions associated with vitamin B12 insufficiency (33). Therefore, supplementation of folic acid should be taken with caution in SCD patients.

Over half of SCD patients in our study had anemia with decreased Hb, but only 36.9% showed typical macrocytosis. It was reported that iron deficiency anemia was presented in about half of CAAG patients due to impaired iron absorption and would precede the appearance of macrocytic anemia, especially in young female patients (34). Therefore, a large number of patients with B12 deficiency would not present as typical megaloblastic anemia accompanied by iron deficiency.

Gastric atrophy is essentially restricted to the gastric corpus and fundus in CAAG. Significantly reduced PG1, PG1/2 ratio and markedly elevated gastrin were characteristic in CAAG patients (35, 36). More studies found that PG1 and PG1/2 ratio, especially the latter, had a significant value in screening for atrophic gastritis and histological staging (37, 38). Therefore, serum pepsinogen would be considered for serological biopsy for gastric atrophy (14, 16). Gastrin, at a high level, was considered as an indicator of decreased acid secretion caused by destruction of parietal cells and loss of feedback inhibition of gastrin secretion (35, 36). However, we did not analyze gastrin due to unavailable data in a number of patients. Future studies should pay more attention on gastrin test. Endoscopy and correct biopsy sampling are essential for diagnosing and distinguishing CAAG (39), but as an invasive and time-consuming examination, gastroscopic examination was undertaken by no more than half of patients in our study, and only nine patients had biopsy. Without biopsy, endoscopic findings are not sufficient to diagnose CAAG because of their low sensitivity and specificity and inter-observer variability (14). Serum pepsinogen would thus be tested as a supplementation or even substitute for evaluating gastric atrophy. Compared to patients with PG1/2 ratio ≥3.0, those with PG1/2 ratio <3.0 had lower B12 level, higher Hcy and MCV, higher rate of absent SEP, and spinal lesions indicating that PG1/2 ratio was associated with the severity of B12 deficiency and hematological and neurological involvement. The inverse correlation of PG1 and PG1/2 ratio with MCV and N20 latency further supported the value of PG1 and PG1/2 ratio in predicting the severity of hematological and neurological involvement. However, patients with CAAG have increased risk to develop gastric neoplasms and other cancers throughout the body (40, 41), and some patients are accompanied by *H. pylori* infection (36). Therefore, regular endoscopic examination is still necessary for SCD patients with CAAG.

When choosing sex- and age-matched ALS patients as controls, the Hcy, folate, RBC count, Hb, MCV, PG1, PG2, and PG1/2 ratio all differed in SCD patients compared to ALS patients. The PG1, PG1/2 ratio had a similar diagnostic accuracy compared to Hcy and MCV (AUC = 0.7–0.8), with very good specificity but lower sensitivity. The combined diagnostic accuracy of RBC, Hcy, and PG1/2, which were the independent risk factor of SCD in our study, had even better diagnostic accuracy as 0.803. PG1/2 ratio <2.4 or <3.0 as cutoff both have high specificity in distinguishing SCD from other non-B12 deficiency diseases, but the PG1 value should be much lower (<22.4 ng/ml) than the generally defined 70 ng/ml to get a relatively good diagnostic accuracy. Thus, low PG1/2 and very low PG1 levels are helpful for diagnosing SCD with high specificity.

There have been no unified diagnostic criteria for SCD so far, but generally low serum vitamin B12 level, elevated Hcy, and increased MCV were considered as part of the diagnostic criteria from laboratory evidences. Based on the evidence in this study, we suggest that low PG1/2 ratio would be considered as a complementary criterion for diagnosing SCD, and prospective cohort studies are needed to verify this.

When a low PG1/2 ratio was found in SCD patients, further examinations such as PCA, anti-IF, gastroscopy, and biopsy are necessary for identifying the existence of CAAG. When SCD coexistence with CAAG is verified, parenteral supplementation of B12 is recommended to attain rapid and optimal correction, and intramuscular—other than oral—vitamin B12 treatment should always be given as maintenance treatment (24).

This study has some limitations. Firstly, the large proportion of patients taking B12 treatment before admission could interfere with the value of serum B12 and related changes in Hcy and hematological system. However, the level of PG1 and PG1/2 ratio related to gastric atrophy could hardly be influenced by B12 treatment. Secondly, instead of healthy controls, we enrolled patients of ALS, which was another neurological disease not due to B12 deficiency, as controls to investigate whether there was a difference in serum pepsinogen between patients of neurological disease with or without B12 deficiency. Strictly speaking, the results of the ROC curve were only fit for comparing SCD and ALS patients. Health controls should be included in future prospective studies. Thirdly, based on the limited proportion of patients undergoing gastroscopy and biopsy, we could not provide direct evidence to identify the correlation of PG with CAAG. Although the high proportion of positive anti-IF and low level of PG1 and PG1/2 ratio would indicate the presence of CAAG, gastroscopy and biopsy would better verify this as a gold standard. Lastly, this was a retrospective study; a variety of biases were hard to be avoided. Prospective cohort studies should be conducted with more rigorous inclusion and exclusion criteria.

In conclusion, in our study, SCD due to B12 deficiency was mostly attributed to CAAG. The significantly decreased serum PG1 level (<22.4 ng/ml) and decreased PG1/2 ratio (<2.4 or 3.0), as serological biomarkers for gastric atrophy, are helpful in distinguishing SCD from other non-B12 deficiency neurological diseases and can reflect the severity of B12 deficiency as well as the severity of hematological and neurological involvement. Serum PG estimation would complement the conventional laboratory parameters for SCD. Prospective cohort studies are needed to further verify this.

## Data Availability Statement

The raw data supporting the conclusions of this article will be made available by the authors, without undue reservation.

## Ethics Statement

The studies involving human participants were reviewed and approved by the Ethics Committee of Chinese PLA General Hospital. Written informed consent for participation was not required for this study in accordance with the national legislation and the institutional requirements.

## Author Contributions

XC and RW: manuscript preparation, data acquisition, and data analysis. XH: data analysis, manuscript editing, and manuscript review. FY: data acquisition and data analysis. SY: concept, design, data analysis, manuscript editing, and manuscript review. All authors contributed to the article and approved the submitted version.

## Conflict of Interest

RW was employed by the General Hospital of Taiyuan Iron and Steel (Group, Co., Ltd.). The remaining authors declare that the research was conducted in the absence of any commercial or financial relationships that could be construed as a potential conflict of interest.
